# How palladium inhibits CO poisoning during electrocatalytic formic acid oxidation and carbon dioxide reduction

**DOI:** 10.1038/s41467-021-27793-5

**Published:** 2022-01-10

**Authors:** Xiaoting Chen, Laura P. Granda-Marulanda, Ian T. McCrum, Marc T. M. Koper

**Affiliations:** 1 Leiden Institute of Chemistry, Leiden University, PO Box 9502, 2300 RA Leiden, the Netherlands; 2 Present Address: Department of Chemical and Biomolecular Engineering, Clarkson University, Potsdam, NY USA

**Keywords:** Electrocatalysis, Fuel cells, Electrocatalysis

## Abstract

Development of reversible and stable catalysts for the electrochemical reduction of CO_2_ is of great interest. Here, we elucidate the atomistic details of how a palladium electrocatalyst inhibits CO poisoning during both formic acid oxidation to carbon dioxide and carbon dioxide reduction to formic acid. We compare results obtained with a platinum single-crystal electrode modified with and without a single monolayer of palladium. We combine (high-scan-rate) cyclic voltammetry with density functional theory to explain the absence of CO poisoning on the palladium-modified electrode. We show how the high formate coverage on the palladium-modified electrode protects the surface from poisoning during formic acid oxidation, and how the adsorption of CO precursor dictates the delayed poisoning during CO_2_ reduction. The nature of the hydrogen adsorbed on the palladium-modified electrode is considerably different from platinum, supporting a model to explain the reversibility of this reaction. Our results help in designing catalysts for which CO poisoning needs to be avoided.

## Abstract (web summary)

Understanding how palladium inhibits CO poisoning is important for rational design of enhanced catalysts. Here the authors show high formate coverage on the palladium-modified electrode inhibits poisoning during formic acid oxidation and the adsorption of CO precursor dictates the delayed poisoning during CO2 reduction.

## Introduction

Low-temperature fuel cells consuming organic molecules as fuel have been regarded as a prospective solution to reduce our dependence on traditional fossil fuels^1,2^. Formic acid is one of the fuel candidates to be employed in a so-called direct formic acid fuel cell (DFAFC)^2,3^. The electrocatalytic formic acid oxidation reaction has also been considered as a model reaction for the oxidation of more complex organic molecules^4^. Of all pure metal electrodes, platinum and palladium show the highest formic acid oxidation activity. Formic acid oxidation on Pt surfaces has been studied extensively and the dual-pathway mechanism^5^ has been well established by the community^2^. This mechanism assumes that there are two parallel pathways in the reaction scheme. One pathway leads to the desired final product CO_2_ at relatively low potentials through a reactive intermediate (presumably some form of adsorbed formate^6^), and another pathway includes a chemical dehydration step leading to adsorbed CO, which acts as a poison blocking the surface and impedes further oxidation of formic acid. The identification of CO as the poisoning intermediate and its role in the oxidation mechanism has been widely accepted^7^, but the nature of the reactive intermediate in the direct pathway is still under strong debate. The prominence of the CO poisoning pathway on Pt electrodes renders Pt an unsuitable catalyst for direct formic acid fuel cells and also not ideal for the study of the direct pathway^8,9^.

Recent advances in catalyst development have led to the synthesis of Pd-based metal nanoparticles with excellent catalytic properties towards formic acid oxidation^10–15^. Pd-based catalysts for electrochemical formic acid oxidation generally display high activity and, remarkably, the absence of CO poison formation. Therefore, Pd model electrodes can be used to study the mechanism of the direct pathway without the interference of CO poisoning, and, perhaps more importantly, to understand how CO poisoning can be avoided. However, Pd single crystals are difficult to prepare. Epitaxially grown Pd layers on a foreign metal are an interesting alternative, particularly Pt single-crystal surfaces modified by a Pd monolayer^10,11,16–20^. The lattice parameters of both metals are close and it has been pointed out that the reactivity of Pd monolayer system is comparable to that of the corresponding Pd single crystal^21^.

Palladium-based materials have also emerged as the best catalysts for the reverse reaction, i.e., carbon dioxide electroreduction to formic acid^22–26^. Theoretically, for a two-electron transfer reaction, such as the conversion between formic acid and CO_2_, reversible catalysts with very low overpotential must exist^27^. In biological systems, Armstrong and Hirst^28^ have discussed how redox enzymes can reversibly catalyze the reaction (HCOOH ⇆ CO_2_ + 2H^+^ + 2e^−^). This raises the question of whether the mechanisms for formic acid oxidation and carbon dioxide reduction on palladium electrocatalysts are similar, i.e., whether they involve similar key intermediates and similar mechanisms for inhibiting CO poisoning.

This opens up the possibility of using palladium-based catalysts for application in unitized regenerative fuel cells based on carbon dioxide and formic acid. Recent efforts from our group have verified that Pd overlayers deposited on polycrystalline Pt, reduce CO_2_ to formic acid and may perform as reversible catalysts^22^. Furthermore, Pd_x_Pt_1-x_ nanoparticles were applied as bifunctional electrocatalysts for both the CO_2_ reduction and formic acid oxidation and showed improved tolerance to CO poisoning and lower overpotentials^29^.

In this paper, we perform systematic electrochemical studies of formic acid oxidation and carbon dioxide reduction on Pd monolayer decorated Pt single crystals and explore the important role played by the involved formate anions on the direct formic acid oxidation pathway. We also study both reactions in comparison to unmodified Pt single crystals. In combination with first-principles density functional theory calculations, our studies reveal the crucial role of adsorbed formate anions in inhibiting CO poisoning during formic acid oxidation. On the other hand, CO poisoning does occur during CO_2_ reduction, but only at relatively high overpotential. Our DFT calculations indicate that the faster poisoning of Pt(111) during CO_2_ reduction is related to the much stronger binding of the key *COOH intermediate on Pt(111) compared to palladium.

## Results

### Formic acid oxidation

**Fig. 1:**
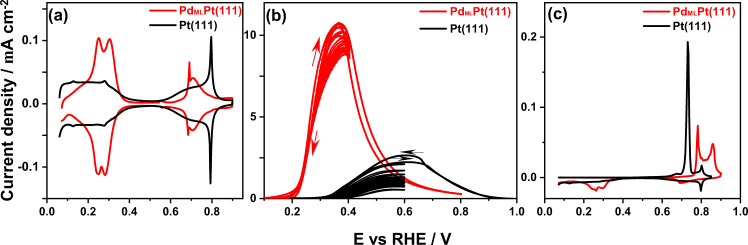
Voltammetry of Pt(111) and Pd_ML_Pt(111). **a** Cyclic voltammogram of Pd_ML_Pt(111) electrode (red) and Pt(111) (black) in 0.1 M HClO_4_. **b** Voltammograms for the oxidation of formic acid on Pd_ML_Pt(111) electrode (red) and Pt(111) (black) in 0.1 M HClO_4_ + 50 mM HCOOH. The evolution of 12 cycles on a rotating Pd_ML_Pt(111) (red) and Pt(111) (black) electrode at 1600 rpm with continuous cycling to two lower vertex potentials: one to the potential of the oxidation peak where no oxidative stripping of adsorbed CO takes place (see panel **c**), and one to a higher potential at which adsorbed CO is oxidatively stripped off. Scan rate: 50 mV s^−1^. **c** CO stripping voltammogram for Pd_ML_Pt(111) electrode (red curve) and Pt(111) (black curve) in 0.1 M HClO_4_. Scan rate: 10 mV s^−1^.

Figure 1a shows the blank voltammogram of Pd_ML_Pt(111) in 0.1 M HClO_4_, compared to Pt(111). The Pd_ML_Pt(111) electrode exhibits the same characteristic regions as Pt(111). For Pt(111), these windows correspond to the H adsorption-desorption feature (0.05 < E < 0.35 V_RHE_), the double-layer region (0.35 < E < 0.60 V_RHE_), and the adsorption-desorption process for OH_ads_ (0.60 < E < 0.90 V_RHE_)^30^. However, as we have shown recently, for the Pd_ML_Pt(111) electrode, these regions correspond to different reactions^31^. The two peaks in the “hydrogen region” of Pd_ML_Pt(111) involve the replacement of adsorbed H by adsorbed OH (peak at E = 0.21 V_RHE_) and the replacement of adsorbed OH by adsorbed ClO_4_^−^ (peak at E = 0.31 V_RHE_). At higher potential (>0.65 V_RHE_), the adsorbed perchlorate is replaced by a higher coverage of OH_ads_ or by adsorbed O. The primary reason for the strong difference between Pd_ML_Pt(111) and Pt(111) surface is the significantly stronger anion and OH adsorption on the Pd_ML_Pt(111) surface.

In Fig. 1b, the voltammograms for the oxidation of formic acid on Pd_ML_Pt(111) and Pt(111) in 0.1 M HClO_4_ containing 50 mM HCOOH are shown. In perchloric acid, the effect of anion adsorption should be minimal, though we do note that we assume specific perchlorate adsorption on Pd_ML_Pt(111) above 0.30 V_RHE_ (see the previous paragraph). As shown in Fig. 1b, on the Pt(111) electrode formic acid oxidation starts from 0.35 V_RHE_ along with a peak current of 2.2 mA cm^−2^ during the positive-going scan, with a slightly higher peak current density at 0.60 V during the negative-going scan because the CO poisoning intermediate has been oxidatively stripped at potentials above 0.70 V_RHE_ (as shown in Fig. 1c). Figure 1b also shows the fast deactivation of the formic acid oxidation on Pt(111) due to the accumulation of the surface-adsorbed CO generated if we cycle to a vertex potential of 0.6 V_RHE_ at which the adsorbed CO is not oxidatively stripped. The formic acid oxidation current decreases fourfold after 12 cycles. These results are consistent with previous results for Pt(111), namely, there exist two parallel pathways (direct and CO formation pathway) during the positive-going scan, while the negative-going scan after CO has been oxidatively stripped at high potential is usually chosen as representative for the formic acid oxidation through the direct pathway only^32^.

For the Pd_ML_Pt(111) electrode, a peak current density of 11.0 mA cm^−2^ at 0.38 V_RHE_ (ca. four times higher current than on Pt(111), at a 0.20 V lower potential) is observed together with a low onset potential at around 0.20 V_RHE_. The remarkable observation in Fig. 1b is that there is hardly any hysteresis for the oxidation current in the positive- and negative-going scan of Pd_ML_Pt(111) electrode between 0.05 and 0.40 V_RHE_, suggesting the absence of CO poisoning. Therefore, only the formic acid oxidation direct pathway occurs, in agreement with previous studies^10,18^. Figure 1c shows that surface-adsorbed CO on the Pd_ML_Pt(111) electrode cannot be oxidatively stripped until the positive-going scan reaches 0.90 V_RHE._ Comparison of the blank voltammograms of Pd_ML_Pt(111) and Pt(111) in Fig. 1a with the formic acid oxidation curves in Fig. 1b indicates that the onset of formic acid oxidation appears to coincide with hydrogen desorption at 0.20 V_RHE_, suggesting that the adsorbed hydrogen inhibits the formic acid oxidation at low potential.

Figure 1c shows the CO stripping voltammograms of Pd_ML_Pt(111) and Pt(111) surfaces in 0.1 M HClO_4_, respectively. Very low currents are measured on both Pd_ML_Pt(111) and Pt(111) electrodes during the positive-going scan until 0.6 V_RHE_, implying that both surfaces are completely blocked by adsorbed CO at low potential. From this observation, we conclude that CO binds strongly and irreversibly to both surfaces. For Pt(111), the oxidative stripping peak for adsorbed CO is located at about 0.72 V_RHE_ and the subsequent scan shows the well-known butterfly feature of Pt(111) in 0.1 M HClO_4_. By comparing to Pt(111), the CO stripping peak of Pd_ML_Pt(111) electrode is shifted to more positive potentials, between 0.80 and 0.90 V_RHE_, suggesting slower CO oxidation kinetics on Pd_ML_Pt(111) compared to Pt(111) under identical experiment conditions, in agreement with a previous report^33^. The charge corresponding to the CO stripping peak is related to the CO coverage, from which we estimate the coverages of CO on Pt(111) and Pd_ML_Pt(111) electrode to be 0.69 and 0.75 ML, respectively, consistent with previous reports for Pt(111)^34,35^ and Pd(111)^36^. From these observations, we infer that we cannot ascribe the lack of CO poisoning during formic acid oxidation on Pd_ML_Pt(111) to a lower CO adsorption strength; if anything, the stripping results show that CO binds stronger to Pd_ML_Pt(111) than to Pt(111).

### Formate adsorption isotherm

In view of the important role of adsorbed formate in the formic acid oxidation process, we determine the adsorbed formate coverages as a function of potential for Pt(111) and Pd_ML_Pt(111) electrodes. At conventional scan rates, the electrochemical signal of formate adsorption is concealed by the formic acid oxidation current. By employing very high-scan rates, it is possible to separate the two processes. The current due to the reversible formate adsorption/desorption process is proportional to the scan rate, whereas the current for the oxidation of formic acid is independent of the scan rate since it is purely kinetically controlled. Consequently, by applying sufficiently fast scan rates, the current corresponding to the adsorption of formate should be much larger than the current corresponding to its oxidation, so that we can neglect the latter contribution^32^.

**Fig. 2:**
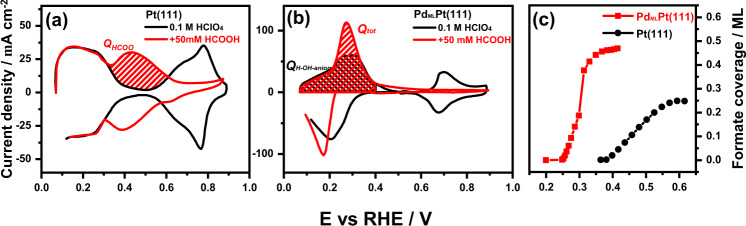
Determination of formate coverage on Pt(111) and Pd_ML_Pt(111). Voltammograms of **a** Pt(111) and **b** Pd_ML_Pt(111) electrode in 0.1 M HClO_4_ (black line) and 0.1 M HClO_4_ + 50 mM HCOOH (red line) solution at a high-scan rate of 50 V s^−1^. **c** Comparison between the coverage of adsorbed formate as a function of potential on the Pd_ML_Pt(111) and Pt(111) electrode.

Figure S2a, b in the Supporting Information shows the blank voltammograms of Pt(111) and Pd_ML_Pt(111) in 0.1 M HClO_4_ solution recorded at 0.05 and 50 V s^−1^, respectively. As can be seen, the currents associated with the typical H_upd_ region, the double-layer region and the OH adsorption region of the Pt(111) electrode in perchloric acid media recorded at 50 V s^−1^ have increased 3 orders of magnitude in comparison to that of 0.05 V s^−1^, as expected. Although there appears to be a change in shape in the low potential region (0.05 < E < 0.40 V_RHE_) of Pd_ML_Pt(111) recorded at 50 V s^−1^ compared to the blank at 0.05 V s^−1^, the charge associated with the H_upd_ and anion adsorption has the same value of 240 µC cm^−2^. The OH adsorption profile of the Pd_ML_Pt(111) electrode, between 0.65 and 0.80 V_RHE_, recorded at 50 V s^−1^ also increases 3 orders of magnitude compared to that recorded at 0.05 V s^−1^. In the presence of formic acid, as shown in Fig. 2a, the high-scan-rate voltammogram of Pt(111) is practically symmetric through the *j* = *0* axis, which indicates that currents are mainly due to adsorption processes and that the contribution from the continuous formic acid oxidation process can be neglected. The characteristic H adsorption-desorption feature between 0.05 and 0.35 V_RHE_ is similar to what is found in 0.1 M HClO_4_, the signal corresponding to formate adsorption is observed between 0.38 and 0.65 V_RHE_, whereas the OH adsorption feature has diminished due to the blocking effect of adsorbed formate. The adsorption process between 0.38 and 0.65 V_RHE_ recorded at 50 V s^−1^ is exclusively caused by formate anion adsorption and shows an anion concentration dependence (as shown in Fig. S2c), and in general, a formic acid containing electrolyte with a higher concentration than 10 mM should be applied to avoid effects of mass-transport limitation during fast-scan-rate technique. All results here are in good agreement with previous fast voltammetry studies^32,37^. The potential region of adsorbed formate agrees with that observed by ATR-FTIR on polycrystalline Pt electrodes^38^.

Figure 2b shows the high-scan-rate voltammogram of Pd_ML_Pt(111) in 0.1 M HClO_4_ + 50 mM HCOOH. On the Pd_ML_Pt(111) surface, the formate adsorption (*Q*_*HCOO*_) starts at 0.20 V_RHE_ in a competitive process with the desorption of hydrogen (*Q*_*H*_). The completion of the formate adlayer is attained in a much narrower and lower potential window compared to Pt(111). The characteristic H feature between 0.05 and 0.30 V_RHE_ overlaps with the signal corresponding to formate adsorption (also see Fig. S2d), whereas we expect that the OH-anion (0.30 < E < 0.40 V_RHE_) and OH/O adsorption (0.60 < E < 0.85 V_RHE_) features would diminish due to the formate blocking effect. To determine the charge corresponding to the adsorption of formate (*Q*_HCOO_) on the Pd_ML_Pt(111) electrode between 0.05 and 0.30 V_RHE_ (see Fig. 2b), we determine the total charge corresponding to the adsorption states in the presence of formate (*Q*_*tot*_), and subtract the charge corresponding to the feature H (*Q*_*H*_) in the absence of formic acid between 0.05 and 0.30 V_RHE_ (see Fig. 2b). *Q*_*H*_ is determined by the known hydrogen coverage (2/3 ML) at the threshold of hydrogen evolution, i.e., at ca. 0.08 V^31^. If the double-layer capacity is the same in the absence and presence of formic acid, we can obtain the experimental isotherms for formate absorption on both surfaces from high-scan-rate voltammetry, assuming an electrosorption valency equal to −1. The formate coverage obtained in this way is the maximum possible coverage of 0.45 ML at 0.40 V_RHE_. Since we do not know if OH or perchlorate is co-adsorbed with formate between 0.30 < E < 0.40 V_RHE_, a minimum coverage can be obtained by subtracting the full blank charge (*Q*_*H-OH-anion*_ in Fig. 2b).

Figure 2c compares the formate coverage-electrode potential curves for the Pt(111) and Pd_ML_Pt(111) electrodes. On Pd_ML_Pt(111), formate adsorbate reaches a saturation coverage of ca. 0.33 (minimum) to 0.45 ML (maximum) at 0.40 V_RHE_, compared to 0.25 ML on Pt(111) at 0.65 V_RHE_. The difference in formate adsorption behavior is ascribed to the anion affinity of Pd surface, which is caused by the difference between the work functions of Pt(111) and Pd_ML_Pt(111)^31^. The result here agrees with a surface-enhanced infrared spectroscopy study, which indicates the band of bidentate formate can be detected from a potential of 0.20 V_RHE_ on Pd electrode in 0.1 M HClO_4_ + 0.5 M HCOOH^39–42^. It is now generally agreed that the adsorbed formate species exist stably on the surface in bidentate form, and does not desorb oxidatively as CO_2_^7,43–46^. Therefore, the bidentate formate should be considered as a spectator species in the formic acid oxidation pathway. A fully saturated layer of bidentate formate has a coverage of 0.5 ML on a per Pt surface atom basis.

### Formate and CO production from CO_2_ reduction

**Fig. 3:**
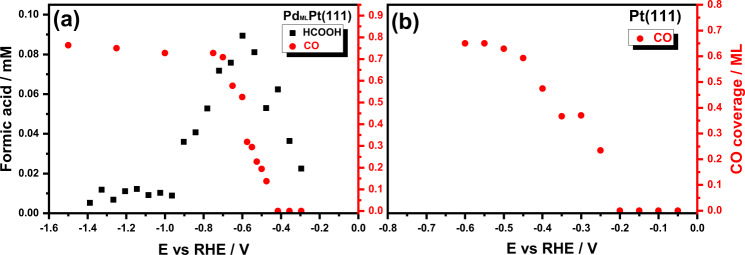
Formate and CO formation. Formation of formic acid detected with online HPLC and the CO coverage calculated from stripping voltammograms on **a** Pd_ML_Pt(111) and **b** Pt(111) electrode under the same experimental condition.

Next, we turn our attention to a comparison of CO_2_ electroreduction on Pd_ML_Pt(111) and Pt(111). Figure 3a, b shows the production of formic acid and (adsorbed) CO from the reduction of CO_2_ on Pd_ML_Pt(111) (Fig. 3a) and Pt(111) (Fig. 3b) electrode as a function of potential. The formic acid production was followed with online HPLC as introduced in the Experimental Section. Figure 3a shows the production of formic acid on the Pd_ML_Pt(111) electrode starts at a potential of −0.29 V_RHE_ and approaches a peak production around −0.60 V_RHE_, and the trend here is similar to our previous results of Pd_x_Pt_(1-x)_ nanoparticles^22^. Recently reported Pd catalysts also demonstrate formate formation at low overpotential and high efficiency^23^. On the other hand, Pt(111) does not produce any measurable amounts of formic acid. Previous studies have shown both Pt(111)^43^ and Pd(111)^44^ single-crystal electrodes to be inefficient CO_2_ electroreduction catalysts as they convert CO_2_ to adsorbed CO as the major product.

To investigate the formation of CO during CO_2_ reduction on the Pd_ML_Pt(111) and Pt(111) electrodes, experiments were carried out by stripping off adsorbed CO by going to positive potentials. We first scanned the potential to different negative vertex potentials performing CO_2_ reduction at 1 mV s^−1^ following the same process as with online HPLC. Next, the electrode was scanned to positive potentials at 10 mV/s, in the same cell immediately after finishing CO_2_ reduction to avoid any contamination/exposure to air during electrode transfer. The corresponding cyclic voltammograms are shown in the Supporting Information (Fig. S4a, b). From the CO stripping charges, we can determine the CO surface coverage generated during the CO_2_ reduction; these CO coverages are shown as red data points in Fig. 3.

Figure 3 shows that on the Pd_ML_Pt(111) electrode CO_2_ reduction starts producing adsorbed CO at potentials more negative than −0.475 V_RHE_, whereas on Pt(111) adsorbed CO is formed at potentials more negative than −0.25 V_RHE_. On Pd_ML_Pt(111), the CO coverage saturates at ca. −0.70 V_RHE_. Compared to the production of formic acid in Fig. 3a, it is also clear that the formation of formic acid drops as the Pd_ML_Pt(111) electrode becomes saturated with adsorbed CO. This is thus a clear indication that the Pd_ML_Pt(111) electrode is able to reduce CO_2_ to formic acid at low overpotential window but becomes passivated due to the formation of a CO adlayer when the potential is more negative.

### DFT results

To better understand the experimental observations during formic acid oxidation and electrochemical CO_2_ reduction on Pd_ML_Pt(111) compared to Pt(111), we used DFT to calculate the adsorption energies of the adsorbates involved in the reactions, *H, *OCHO, *COOH, *CO. We will focus on explaining the absence of *CO poisoning on Pd_ML_Pt(111) during formic acid oxidation at low potentials, in comparison to the rapid poisoning on Pt(111) (see Fig. 2), based on the adsorption characteristics of formate *OCHO. In the case of the CO_2_ reduction reaction, we will provide a thermodynamic explanation of the rapid CO poisoning of Pt(111) vs Pd_ML_Pt(111) based on the adsorption energetics of *COOH, the precursor of *CO, on both surfaces.

**Fig. 4:**
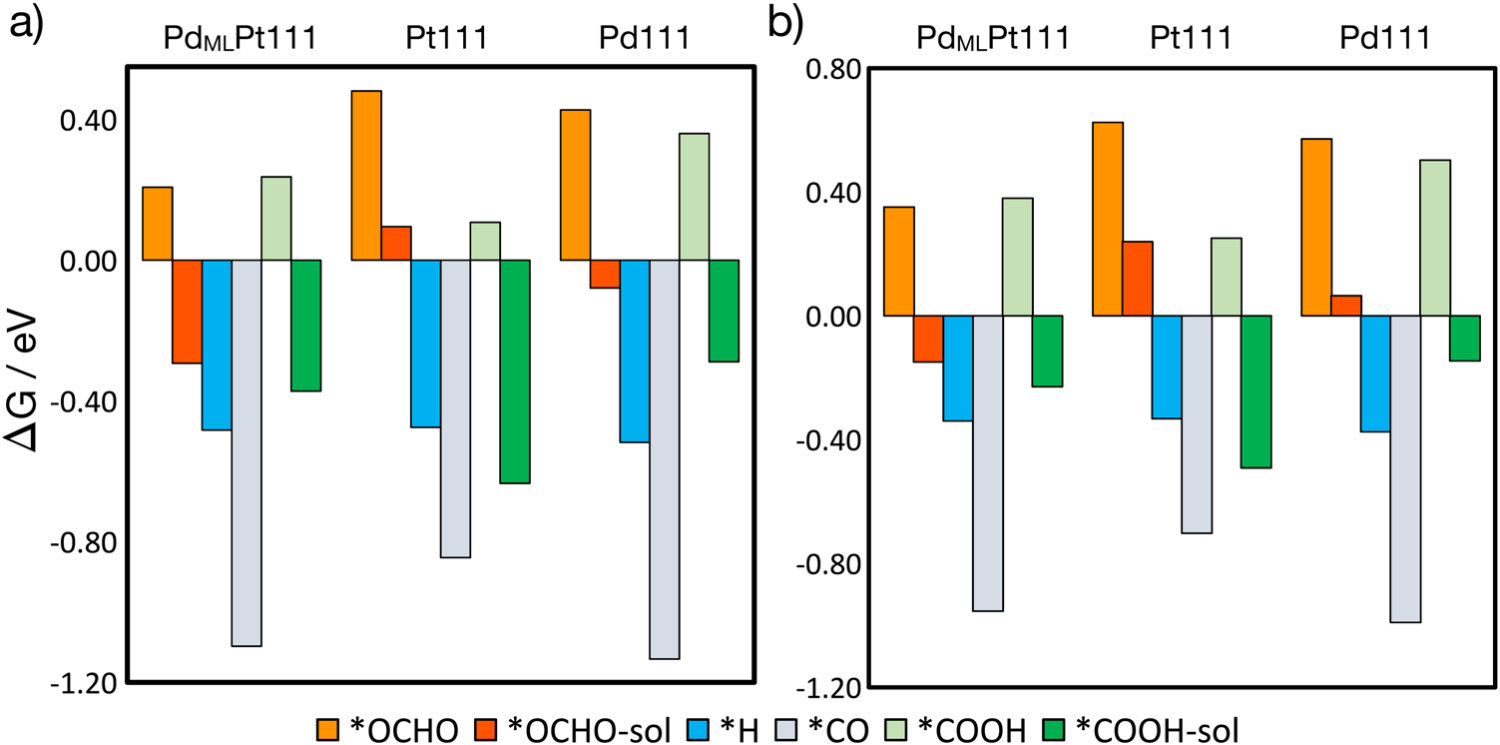
Formation energies of various adsorbates. **a** Formation energies of adsorbed *OCHO, *H, *COOH, and *CO at 1/9 ML coverage at 0 V vs RHE on Pd_ML_Pt(111), Pt(111), and Pd(111) from formic acid in solution. **b** Formation energies of adsorbed *OCHO, *H, *COOH, and *CO at 1/9 ML coverage at 0 V vs. RHE on Pd_ML_Pt(111), Pt(111), and Pd(111) calculated relative to CO_2_ (g), electrons, and protons, and for *H, calculated relative to (H^+^ + e^−^). *OCOH in bright orange is non-solvated and in dark orange/red it is solvated, *OCHO-sol. *COOH in light green is non-solvated and in dark green it is solvated, *COOH-sol.

Figure 4a shows the free energies of adsorption/formation of *H, *CO, *OCHO, *COOH at 1/9 ML coverage at 0 V vs RHE. The free energy of *COOH in the absence of solvation is in light green and with solvation, *COOH-sol, in dark green, and for *OCHO, bright orange is without solvation and dark orange/red is with solvation. The energies shown in Fig. 4a are calculated from formic acid in the solution phase. For solvated *COOH, we used *COOH with two water molecules, one of which is hydrogen-bonded to the OH in *COOH, and for solvated formate, we used 1 H_2_O molecule; for more details about solvation, see the Supporting Information. The most favorable adsorption sites on all electrode surfaces for *H and *CO were the highly coordinated fcc hollow sites. Note that for Pt(111) we used *CO atop configuration instead, as experimentally this is known to be the preferred site. The difference in energy between atop and fcc for *CO on Pt(111) at 1/3 ML coverage is ~0.1 eV. We found that the most favorable configuration of formate, *OCHO, is bound to the surface of the electrode through the oxygen atoms with each oxygen adsorbed atop a surface atom.

The adsorption trend on Pd_ML_Pt(111) and Pd(111) is *OCHO-sol < *COOH-sol < *H < *CO, and on Pt(111) surface, hydrogen has a weaker adsorption than *COOH-sol, and the trend is *OCHO-sol < *H < *COOH-sol < *CO. However, at higher coverages of 1/4 ML, the adsorption energy of *H is more favorable than that of *COOH-sol. Also, formate adsorption is significantly more favorable on Pd_ML_Pt(111) and Pd(111) than on Pt(111), while *COOH-sol adsorption is more favorable on Pt(111) than on Pd_ML_Pt(111) and Pd(111), and this trend holds for both coverages, 0.11 ML and 0.25 ML. Bader charge analysis shows more negative charge is retained on the adsorbates adsorbed on Pd_ML_Pt(111) and Pd(111) than on Pt(111). (See Table S4 in the Supporting Information). We can conceptually explain this formate adsorption and charge trend in terms of the different work functions of the catalysts, where the surface with the lower work function (and hence also a corresponding lower potential of zero charge) is expected to have a higher affinity for anion adsorption. The trend in work function follows Pd_ML_Pt(111) (5.14 eV)<Pd(111) (5.29 eV)<Pt(111) (5.74 eV).

**Fig. 5:**
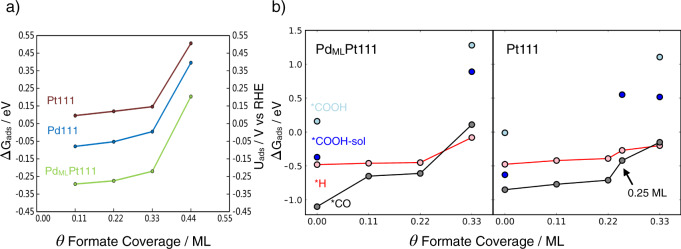
Adsorption energies and potentials of adsorbates. **a** Adsorption/formation energies (left *y*-axis) and corresponding adsorption potentials (right *y*-axis) for adsorbed formate from formic acid in solution as a function of coverage, on Pd_ML_Pt(111) (green), Pd(111) (blue), and Pt(111) (red). **b** Free energy of adsorption of *H, *CO, and *COOH corresponding to 1/9 ML at 0 V vs RHE, for Pd_ML_Pt(111) and Pt(111), as a function of different coverages of co-adsorbed formate from 0.11 to 0.33 ML also including 0.25 ML for Pt(111) as specified with the arrow, because this is the experimentally observed maximum coverage on Pt(111). Only the low and higher coverages for *COOH are shown (0.11 ML and 0.33 ML), where *COOH-sol (blue) is with solvation and *COOH (light blue) is without. Connecting lines are only intended as a guide for the eye.

We further investigated formate adsorption on each surface, by calculating the formate adsorption energy as a function of coverage, see Fig. 5a. We find that formate adsorbs significantly more strongly on Pd_ML_Pt(111) than Pt(111) and Pd(111) at all investigated coverages, in agreement with the experimental results in Fig. 2. At high coverages, beyond 0.33 ML, formate anions can no longer adsorb in a bidentate configuration, and adsorption is significantly less favorable on all three surfaces than adsorption at low coverages.

In the potential region where formic acid oxidation occurs, above 0.2 and 0.4 V for Pd_ML_Pt(111) and Pt(111), respectively (see Fig. 1), formate can favorably adsorb up to relatively high coverages of 0.33 ML, where 2/3 of the surface atoms are blocked. Therefore, we have investigated the effect of this adsorbed formate, from 0.11 to 0.33 ML, on the formation of the adsorbed reaction intermediates, *H, *CO, *COOH-sol from formic acid. The results are shown in Fig. 5b. Figure 5b shows the free energy of adsorption of *H, *CO, and *COOH-sol co-adsorbed with formate at coverages from 0.11 to 0.33 ML on Pd_ML_Pt(111) (left) and Pt(111) (right). The free energy of the solvated *COOH-sol with 0.33 ML of *OCHO, contains an upper bound estimate of the solvation effect on *COOH, as the solvation energy calculated for the non-co-adsorbed system is added to the free energy of the co-adsorbed one. This is less computationally intensive than modelling explicit solvation for the high-coverage co-adsorbed system (see Supporting Information for more details).

For Pd_ML_Pt(111), the adsorption strength of *H is not significantly altered when co-adsorbed with 0.11–0.22 ML of formate, and its adsorption energy is more positive (less favorable) than that of *CO at the same conditions. However, because the adsorption strength of *CO is more strongly affected by the presence of co-adsorbed formate, at formate coverages of 0.33 ML, the trend is reversed and *CO is clearly less favorably adsorbed than *H by ~0.20 eV. Thus, at 0.33 ML formate coverage, it is more favorable for *H to be co-adsorbed with formate than *CO. Furthermore, adsorbed formate at 0.33 ML weakens *COOH-sol adsorption, as seen in Fig. 5b, and consequently hinders its further dissociation to *CO, providing a further reason why CO poisoning is not observed during formic acid oxidation in the experiments on Pd_ML_Pt(111).

For Pt(111), the presence of formate also weakens *H and *CO adsorption but not as significant as on Pd_ML_Pt(111), meaning that the effect of formate on their adsorption strength is smaller on Pt(111). The effect of co-adsorbed formate on the difference in the formation energy of *CO and *H is also smaller on this surface than on Pd_ML_Pt(111). At formate coverages of 0.33 ML, the difference in *H, and *CO adsorption strength is only 0.05 eV. Furthermore, as formate adsorption is weaker on Pt(111) than on Pd_ML_Pt(111), its adsorption potential is more positive (see Fig. 5a), and in general a lower coverage of formate will be adsorbed on Pt(111) than on Pd_ML_Pt(111) at the onset of formic acid oxidation. At a formate coverage of 0.25 ML, the Pt(111) surface is only 50% covered, leaving active sites available for *CO formation from its precursor *COOH-sol, given its favorable formation energy even in the presence of this co-adsorbed formate. This results in *CO poisoning of Pt(111) during formic acid oxidation.

Formic acid oxidation starts at 0.2 V_RHE_ on Pd_ML_Pt(111), and at higher potential, 0.4 V_RHE_, on Pt(111), in agreement with calculated formate adsorption strengths being stronger and weaker, respectively. Thus, formate adsorption is important for formic acid oxidation on both Pd_ML_Pt(111) and Pt(111), not as an active intermediate, but more as a self-protector against CO poisoning. Strong formate adsorption on Pd_ML_Pt(111) results in a high coverage, blocking sites for the formation of the *COOH (the *CO precursor) and weakening the binding of *CO, preventing *CO poisoning on this surface. Also, as the effect of co-adsorbed formate is smaller on other intermediates, such as *H, the oxidation reaction can still proceed rapidly through non-*CO containing paths, for instance where the active intermediate is formate with C–H-down type configuration. However, on Pt(111), the adsorption strength of formate is weaker, yielding a lower adsorbed formate coverage during reaction conditions, leaving empty active sites for the *CO poisoning pathway.

Figure 4b shows the adsorption free energy of *OCHO, *COOH, *CO, and *H, on Pd_ML_Pt(111), Pt(111), and Pd(111) calculated relative to CO_2_ (g), electrons, and protons except for *H, calculated from proton reduction. The adsorption energy trend at 1/9 ML coverage on Pd_ML_Pt(111) and Pd(111) follows *OCHO-sol < *COOH-sol < *H < *CO whereas on Pt it is *OCHO-sol < *H < *COOH-sol < *CO. The main difference in the trends is that on Pt(111) the adsorption strength of *COOH-sol is stronger than that of *H at this coverage. *CO and *OCHO-sol are much more strongly adsorbed on Pd_ML_Pt(111) and Pd(111), but *COOH is much more strongly adsorbed on Pt(111). In the case of hydrogen, its adsorption free energy is similar on all the surfaces.

The observation we make from Figs. 4 and 5b is that under conditions of CO_2_ reduction (i.e., negative potentials), *H is always more stable than *OCHO. This is basically in agreement with our observation from Fig. 1 that *H inhibits formate adsorption. Therefore, we consider that *OCHO is not the intermediate in the CO_2_ reduction to formic acid/formate. There is growing agreement in the literature that for catalysts that reduce CO_2_ close to the thermodynamic potential, including palladium, the key intermediate is *H, i.e., hydride^23,47^. Formate is then formed by nucleophilic attack of the *H to the carbon of CO_2_^23,47^.

It is therefore likely that during the reduction reaction, the surfaces are covered with hydrogen. With this in mind, we investigated the effect of 1/3 and 1 ML hydrogen coverage on the adsorption energetics of *COOH (see Fig. S6), and the chemical nature of the adsorbed hydrogen by calculating partial Bader charges (see Table S4) and the corresponding work function (Table S5) for 1/9, 1/3, and 1 ML coverages, which provides a qualitative analysis of our results.

From Fig. S6 we observe that the *COOH free energy of adsorption becomes less favorable as hydrogen coverage increases due to repulsive interactions, but *COOH remains more favorably adsorbed on Pt(111) than on Pd_ML_Pt(111) and Pd (111). We note that from the experiment (Fig. 3) CO* formation starts 0.25 V earlier on Pt(111), and in Fig. S6, the adsorption energy of COOH* in the presence of 1 ML of H* looks to be roughly 0.25 eV more favorable on Pt(111) than on Pd_ML_Pt(111) and Pd(111).

It is interesting to note that the partial charge of the hydrogen adsorbed on the Pd_ML_Pt(111) and Pd(111) surfaces is much more negative than that on the Pt(111) surface at all coverages. This creates a different trend in dipole moment/work function on the surfaces: an increase on Pd_ML_Pt(111) and Pd(111) and a decrease on Pt(111) up to 1 ML, see Tables S4 and S5 in the Supporting Information. Our DFT results match the trend seen in experimentally measured changes in the work function of Pt(111)^48^ and Pd(111)^49^ in UHV during the adsorption of hydrogen. Although we do not have direct evidence for the exact mechanism to form formic acid, we hypothesize that the significantly different chemical states of the hydrogen on Pd vs. Pt play an important role. Since hydrogen has a more negative partial charge on both Pd(111) and Pd_ML_Pt(111) than on Pt(111), it is expected to act as a reactive hydride species (facilitating a nucleophilic attack to the carbon) to form formic acid^47,50^. On Pd(111) and Pd_ML_Pt(111), the partially negatively charged hydrogen can either be transferred to a non-adsorbed CO_2_ molecule that is very close to the surface, or to the carbon of adsorbed *COOH. This nucleophilic attack is less likely to occur on Pt(111) because the surface hydrogen has a much less negative partial charge. Also, on Pt(111) the *COOH adsorbs more strongly, leading to the surface becoming covered/poisoned with CO* at a less negative potential so that the nucleophilic attack by H* on CO_2_ cannot occur.

On the basis of Fig. 4b, *COOH-sol adsorption is more favorable on Pt(111) than on Pd_ML_Pt(111). The limiting potentials for the formation of *COOH-sol on Pd_ML_Pt(111), Pd(111), and Pt(111) are 0.23, 0.15, and 0.49 V_RHE_. Therefore, the formation of *CO from its precursor *COOH-sol is likely to occur on Pt(111) at less negative potentials compared to Pd_ML_Pt(111) and Pd(111), in good agreement with the experimental observations for Pd_ML_Pt111 and Pt(111) (see Fig. 3). Also note that *COOH-sol needs to replace *H, which is favorable only on Pt(111) (see Fig. 4b). Therefore, *CO formation is more likely to occur on Pt(111) than on Pd_ML_Pt(111) as the adsorption of its precursor *COOH occurs at earlier potentials than on Pd_ML_Pt(111). Once the surface is covered with *CO, *H can no longer form and the pathway to forming formic acid/formate is blocked.

As mentioned in the Introduction, our main reason for studying Pd_ML_Pt(111) instead of Pd(111) is the absence of hydrogen absorption in the former, which considerably simplifies (the interpretation of) the experiment. On the pure Pd(111) surface, we might also expect subsurface or absorbed hydrogen (hydride) to play a role in CO_2_ electroreduction, as hydrogen absorption into pure palladium is favorable under CO_2_ electroreduction conditions^51^. This role could be a direct one, where subsurface hydrogen participates in the reduction mechanism, or an indirect one, where the presence of absorbed hydrogen alters the properties of the palladium surface, altering the thermodynamics and kinetics of CO_2_ reduction. However, as hydrogen absorption has not been observed on the Pd_ML_Pt(111) surface (presumably because there are no “bulk” Pd atoms, only a single Pd monolayer), nor on Pt(111), we have not examined the effects of absorbed hydrogen here. The effects of absorbed hydrogen may alter the behavior of Pd(111) relative to what we have found here on Pd_ML_Pt(111) and Pt(111), as only the Pd(111) surface would be capable of absorbing significant hydrogen subsurface. Still, we note that the high activity of Pd_ML_Pt(111) for CO_2_ electroreduction to formate is very similar to bulk Pd electrodes so that we do expect our conclusions to be transferable.

## Discussion

### Formic acid oxidation

The experimental results and the DFT calculations indicate that adsorbed formate plays a key role in preventing CO poisoning on the Pd_ML_Pt(111) electrode during formic acid oxidation. The DFT calculations predict that the effect of co-adsorbed formate towards weakening CO* is larger on Pd_ML_Pt(111) than on Pt(111), such that at the maximum coverage of formate on Pd_ML_Pt(111), i.e., 0.33 ML, the binding energy of CO is unfavorable compared to H, whereas at the maximum coverage of formate on Pt(111), i.e., 0.25 ML, the binding energy of CO is still more favorable compared to H. Therefore, CO formation from *COOH is suppressed on Pd_ML_Pt(111), but still happens on Pt(111). There is, however, an additional geometric argument why CO poisoning on Pd_ML_Pt(111) would be suppressed. It is well known that the formation of CO from formic acid requires an ensemble site of two (or more) neighboring free sites^7,52^. The Pd_ML_Pt(111) shows strong formate adsorption with high saturation coverage of 0.33 ML. This 1/3 ML formate coverage means that at full coverage, 2/3 of the Pd surface atoms are blocked and the ensemble site of two neighboring Pd sites is not available. Therefore, CO poisoning is inhibited geometrically. On the other hand, the 1/4 ML saturation coverage of formate on Pt(111) is not high enough to block the ensemble site, and hence the Pt(111) surface becomes easily poisoned by CO.

Interestingly, while the formation of *CO is blocked on the 0.33 ML formate-covered Pd_ML_Pt(111) electrode, formic acid oxidation still takes place at a high rate. This can be explained by recent models for formic acid oxidation, which consider adsorbed formate as a “spectator”, and which identify the active formate intermediate as a formate species interacting to the surface through the C–H bond. This configuration with the C–H pointing to the 1/3 ML of “free” Pd_ML_Pt(111) surface sites can react to CO_2_ by fast C–H cleavage due to the affinity of the Pd surface to hydrogen. Note that in this picture, adsorbed formate is more than a spectator, as it specifically blocks the surface from CO formation, and thereby protects the surface from poisoning. On Pt(111), the adsorbed formate does not bind strong enough to play the same role. In more chemically intuitive terms, we attribute this ability of palladium to “self-protect” from CO poisoning to the higher affinity of Pd and Pd_ML_Pt(111) to anions. We relate the higher anion affinity of Pd_ML_Pt(111) and Pd compared to Pt(111) to their lower work function, and hence a lower potential of zero charge.

**Fig. 6:**
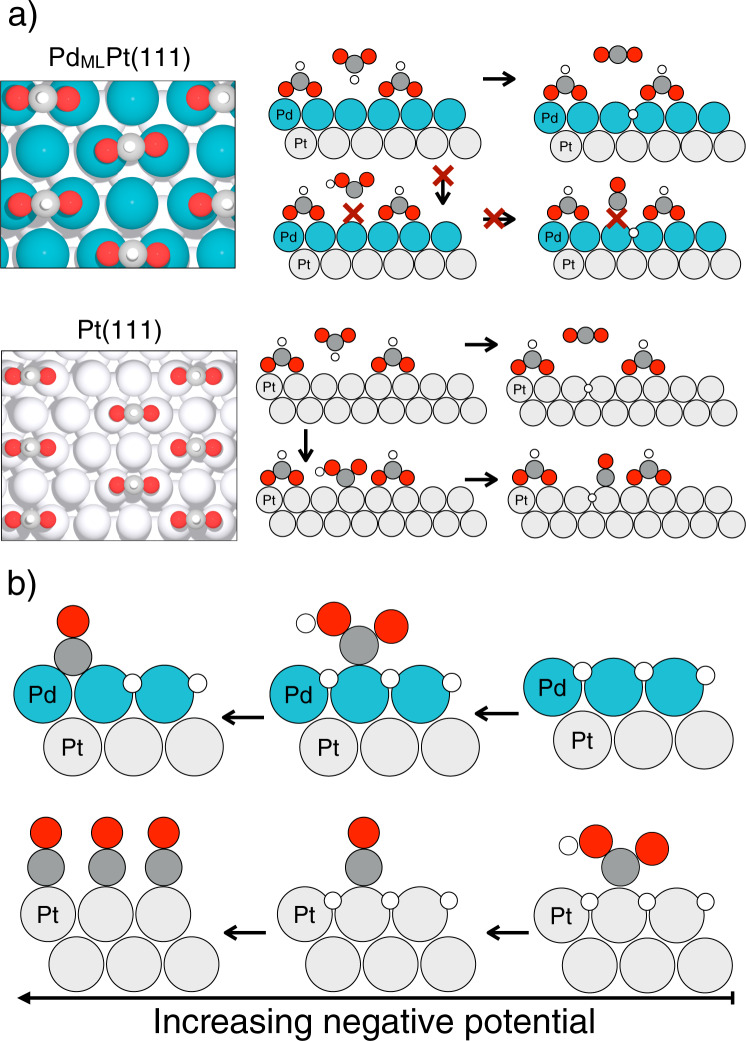
Illustration of the model mechanisms. **a** For the CO poisoning pathway during formic acid oxidation on Pd_ML_Pt(111) (upper drawing) and Pt(111) (lower drawing) with top views of the surfaces on the left and side views on the right. During formic acid oxidation the formate coverage on Pd_ML_Pt(111) is 1/3 ML, and on Pt(111) is 1/4 ML. On Pt(111) the necessary two neighboring surface atoms for *CO formation from *COOH are available, while on Pd_ML_Pt(111) they are not available. **b** For the CO poisoning pathway during CO_2_ reduction reaction, on Pd_ML_Pt(111) (upper sketch) and Pt(111) (lower sketch). Note that *COOH is adsorbed on Pt(111) at a less negative potential than on Pd_ML_Pt(111). Oxygen is in red, carbon in gray, hydrogen in white.

Our model for formic acid oxidation and the mechanism of CO poisoning is illustrated in Fig. 6a. On Pd_ML_Pt(111), the surface is covered with such a high coverage of formate, that the only remaining interaction of formate with the surface is through the C–H bond. This interaction cleaves the C–H bond and releases CO_2_. The pathway to *CO formation is blocked because the ensemble site for CO formation is unavailable and because the binding of CO at such a high formate coverage is highly unfavorable. On Pt(111), the formate coverage is lower. Formate is presumably still activated through cleaving the C–H bond, making the adsorbed formate an inactive spectator species. However, the ensemble site for CO formation is available and the binding of CO at this formate coverage is still reasonably favorable. As a result, the surface will accumulate *CO and becomes poisoned.

We have attempted to test this model for the Pd_ML_Pt(100) electrode, which shows mass-transport-limited formic acid oxidation at a normal scan rate^20^. Figure S3 shows fast voltammetry results for the formic acid oxidation on Pd_ML_Pt(100) electrode. Unfortunately, the oxidation of formic acid on Pd_ML_Pt(100) electrode is still very fast, even in 0.1 M HClO_4_ + 50 mM HCOOH at 50 V s^−1^, so that this electrode is too active to determine the saturation coverage of adsorbed formate, and hence we cannot confirm that on Pd_ML_Pt(100), adsorbed formate protects the surface from CO poisoning. However, we do believe that it is likely that also on other facets of Pd (and hence on polycrystalline Pd) formate adsorbs more strongly than on Pt, and this would explain the higher resistance of Pd towards CO poisoning.

### CO_2_ reduction

Pd surfaces produce formate at low potential close to the thermodynamic potential of formic acid formation from CO_2_ reduction. At more negative potentials, the surface passivates due to the accumulation of CO^22,23^. The electrochemically generated surface-adsorbed hydrogen has been hypothesized to play a key role during the electrohydrogenation of CO_2_ to formate on the Pd surface^23^. In this case, high-coverage formate adsorption cannot explain the absence of CO poisoning at low overpotential, as formate adsorbs only at potentials positive of hydrogen adsorption (see Fig. 2). Therefore, we also consider it unlikely that adsorbed formate is the intermediate in the CO_2_ reduction on palladium. Supporting the hypothesis of the electrochemically generated adsorbed hydrogen being the reactive species, DFT shows that the nature of this adsorbed hydrogen differs significantly from Pd_ML_Pt(111) to Pt(111). On Pd_ML_Pt(111) it is more negatively charged than on Pt(111), and this negatively charged hydrogen (“hydride”) can be transferred to a positively charged carbon (on a CO_2_ molecule close to the surface (or to an adsorbed *COOH)) through a nucleophilic attack, to form formate^47,50^. Another attractive feature of this model for formate formation is that it is, on the molecular level, the exact reverse of how formate is oxidized, namely through the formation/cleavage of a C–H bond with the H interacting with the catalyst surface. It is likely that this molecular reversibility is related to the observed kinetic reversibility of this reaction when carried out on a suitable catalyst.

The DFT calculations suggest that the CO poisoning during CO_2_ reduction is related to the stability of the *COOH intermediate. Since *COOH is considerably more stable on Pt(111) compared to Pd_ML_Pt(111), CO poisoning happens much faster on Pt(111) and formic acid/formate is not produced (see Fig. 3). Our model for CO poisoning during CO_2_ reduction reaction is illustrated in Fig. 6b. Summarizing, on Pt(111) *COOH adsorbs at less negative potentials than on Pd_ML_Pt(111). However, at sufficiently negative potential, *COOH formation becomes favorable on Pd_ML_Pt(111) as well and therefore we start seeing *CO poisoning also on that surface.

In conclusion, we have used a well-defined epitaxially grown Pd monolayer on Pt(111) in comparison to a Pt(111) single-crystal electrode to unveil the detailed relationship between surface structure, adsorbed intermediates, and reactivity for electrocatalytic formic acid oxidation and CO_2_ reduction, with the specific aim to understand the ability of Pd catalysts to withstand CO poisoning and to probe if the mechanisms for oxidation and reduction involve similar intermediates. The Pd_ML_Pt(111) surface shows a higher activity for formic acid oxidation than Pt(111). Our fast-scan voltammetry results show a higher coverage of 1/3 ML formate anion adsorption on the Pd_ML_Pt(111) electrode compared to saturation coverage of 1/4 ML on Pt(111). Supported by DFT results, we argue that the high binding energy of formate and the resulting higher coverage of formate anions, blocks the ensemble site necessary for CO formation, explaining why palladium does not poison by CO during formic acid oxidation. During CO_2_ reduction, the Pd_ML_Pt(111) surface produces formate at a low potential of −0.29 V_RHE_ but starts producing adsorbed CO at potentials more negative than −0.475 V_RHE_, whereas Pt(111) is poisoned at less negative potential and never produces formate. Combined experimental and DFT results suggest that the faster poisoning on Pt(111) compared to Pd_ML_Pt(111) is due to stronger adsorption of *COOH, the precursor of *CO, at less negative potentials on Pt(111). As for the mechanism of the reaction, during CO_2_ reduction, the electrochemically generated adsorbed surface hydrogen is significantly more negatively charged on Pd_ML_Pt(111) than on Pt(111), and as a result, formation of formate via a nucleophilic attack of the negatively charged hydrogen to a positively charged carbon is more likely. This indeed appears as the reverse of the currently accepted mechanism for formic acid oxidation^4^, in which the activation of formate involves the breaking of the C–H bond leading to a (transient) adsorbed hydrogen/hydride intermediate. Therefore, we conclude that the mechanism for the interconversion between formate and carbon dioxide is indeed the same, as would be expected for a reversible reaction, whereas the mechanisms for poisoning are different, presumably because the poisoning happens at higher overpotentials.

## Methods

### Experimental section

#### Electrochemical measurements

Electrolytes were prepared from ultrapure water (Merck Millipore, 18.2 ΜΩ cm, TOC < 3 ppb) and high-purity reagents (Merck Suprapur, Sigma–Aldrich Trace Select). Before each experiment, the electrolytes were first purged with argon (Air Products, 5.7) for 30 min to remove air from the solution. In the case of CO_2_ reduction experiments, the electrolyte was subsequently purged with CO_2_ (Linde, 4.5) for at least 30 min to saturate the solution. For CO stripping experiments, the single-crystal electrode was in contact with a CO (Linde 6.0) saturated solution in hanging meniscus configuration at a fixed potential of 0.1 V_RHE_ for 30 s, which is sufficient to form a complete monolayer of CO on the electrode. Afterwards, argon was bubbled for 15 min to remove CO from the solution, followed by the CO oxidative stripping experiment.

Cyclic voltammetry measurements were carried out in standard electrochemical cells using a three-electrode assembly at room temperature. All glassware was cleaned in an acidic solution of potassium permanganate overnight, followed by rinsing with an acidic solution of hydrogen peroxide and repetitive rinsing and boiling with ultrapure water. Pt(111) and Pt(100) bead-type electrodes were used as working electrodes (diameter of 2.27 and 3.46 mm, respectively) for cyclic voltammetry, and 10 mm disk-type electrodes were used for online high-performance liquid chromatography (HPLC) experiments, resp. Prior to each experiment, the working electrodes were prepared according to the Clavilier method^53^. A platinum wire was used as a counter electrode and a reversible hydrogen electrode (RHE), in a separate compartment filled with the same electrolyte, at the same pH as the electrolyte in the electrochemical cell, was employed as the reference electrode. The electrochemical measurements were performed with the single-crystal electrode in the hanging meniscus configuration. The potential was controlled with an Autolab PGSTAT302N potentiostat. The fast-scan cyclic voltammetry experiments were performed using a Bio-Logic SP-300 potentiostat. The current density shown here represents the measured current normalized to the geometric area of the working electrode.

#### Preparation of Pd monolayers on Pt(111) and Pt(100) single crystals

The Pd monolayers were prepared using a method similar to the one reported before^21,54^. The freshly prepared Pt(111) and Pt(100) electrodes were immersed into a Pd^2+^ containing solution at 0.85 V_RHE_, where no Pd deposition occurred, and the potential was continuously cycled between 0.07 and 0.85 V_RHE_ at 50 mV s^−1^. The amount of palladium on the Pt(111) surface was monitored by following the evolution of the voltammetric peak at 0.23 V_RHE_ (as shown in Fig. S1a), characteristic of the presence of Pd adatoms, whose charge (and current density) depend on the palladium coverage. Scanning tunnelling microscopy (STM) experiments have revealed the existence of small monoatomic high Pd islands which nucleate on the Pt(111) surface with no noticeable preference of nucleation sites and a full Pd monolayer without detectable holes can be formed after deposition^33^. The STM images show the presence of an ordered sulphate adlayer with a (√3×√9)R19.1° structure on the Pd monolayer^33^. After preparation, the Pd_ML_Pt(111) electrode was taken from the cell and thoroughly rinsed with ultrapure water before electrochemical measurements. For the Pd_ML_Pt(100) electrode, the single crystal was taken from the cell and then immersed in a nitrite saturated solution at an open circuit to generate a layer of nitric oxide (NO) on the surface. Next, the crystal was thoroughly rinsed with ultrapure water to avoid any contamination from the acidic nitrite solution and subsequently was transferred to the electrochemical cell at 0.85 V_RHE_ and the adsorbed NO was reductively stripped. The NO procedure is a kind of electrochemical annealing which leads to a Pt(100) electrode fully covered by a single palladium monolayer^54^. The palladium monolayer was monitored by following the evolution of the voltammetric peaks at 0.17, 0.27, and 0.39 V_RHE_ (as shown in Fig. S1b).

#### Online high-performance liquid chromatography (HPLC)

The detection of products dissolved in the electrolyte during CO_2_ reduction as a function of applied potential was performed by online HPLC^55^. Similar to the method reported from our group^29^, samples were collected using an open tip positioned close to the electrode (∼10 μm) while the potential was changed from 0.0 V to the required potential. Sampling was performed at a rate of 60 μL min^−1^, and each sample has a volume of 60 μL. Since the potential was varied at the rate of 1 mV s^−1^, each sample contains the products averaged over a potential change of 60 mV. After voltammetry, the collected samples were analyzed by HPLC (Prominence HPLC, Shimadzu; Aminex HPX 87-H column, Biorad).

#### Computational details

Density functional theory (DFT) calculations were used to compute the adsorption/formation energies of *H, *CO, *OCHO, and *COOH adsorbates involved in the formic acid oxidation and CO_2_ reduction reactions on Pd_ML_Pt(111) (Pt(111) covered by one monolayer of Pd), Pt(111) and Pd(111). All calculations were performed using the PAW^56^ method in the Vienna Ab initio Simulation Package^57^ using the PBE^58^ exchange-correlation functional. We use 3 × 3 unit cell slabs to simulate adsorbate coverages of 0.11–0.33 ML and used 2 × 2 unit cell slabs to investigate adsorbate coverages of 0.25 ML. The Pd_ML_Pt(111), Pt(111), and Pd(111) were simulated with five, six and four atomic layers, respectively. This choice was made based on convergence tests of adsorption energies with the different layers. The k-point samplings for the 3 × 3 unit cell slabs were 6 × 6 × 1 for Pd_ML_Pt(111) and Pt(111) and 4 × 4 × 1 for the Pd(111); for the 2 × 2 unit cell slabs, 6 × 6 × 1 for Pd(111) and 4 × 4 × 1 for Pd_ML_Pt(111) and Pt(111). The two bottommost layers were fixed at the calculated lattice constant of the bulk metal, 3.98 Å for Pt(111), also used for the Pd_ML_Pt(111) slab, and 3.93 Å for Pd(111), and the remaining atomic layers were relaxed. The method of Methfessel-Paxton^59^ to the second-order was used to set the partial occupancies on each orbital and the smearing width was set to 0.2 eV for surfaces and adsorbed species. For the individual molecules H_2_ (g), H_2_O(g), CO_2_ (g), CO (g) a Gaussian smearing of 0.001 eV was used instead, and they were calculated in an asymmetric box of (15.0 × 15.1 × 15.3) Å at a k-point sampling of 1 × 1 × 1. The maximal forces on the atoms were converged to 0.02 eV Å^−1^ for all simulations and the plane wave cutoff was set to 450 eV. A vacuum spacing of ~15.0 Å between metal slabs was set to simulate the surfaces of Pd_ML_Pt(111), Pt(111), and Pd(111), and dipole corrections were also applied in the surface normal direction. The free energy of H_2_O (g) was corrected to the free energy of H_2_O (l) by adding −0.0887 eV to the TS term^60^. The adsorption/formation free energies of *H, *CO, *OCHO, and *COOH were calculated from formic acid in solution, (HCOOH (aq)) for the formic acid oxidation reaction, and from carbon dioxide in gas-phase, (CO_2_ (g)), protons and electrons for the CO_2_ reduction reaction. Gas-phase energy corrections for CO_2_(g) and CO (g) are included^61^, and the CHE model^60^ is used for the coupled proton and electron transfer. The exact details for calculating the (free) adsorption energies of the different species from the DFT energies are outlined in the Supporting Information.

## Supplementary information

Supporting Information

## Data Availability

The datasets generated and/or analyzed during the current study are available from the corresponding author on reasonable request.
