# Supplementary material for: How palladium inhibits CO poisoning during electrocatalytic formic acid oxidation and carbon dioxide reduction
Source: Nat Commun. 2022 Jan 10;13:38. doi: 10.1038/s41467-021-27793-5 (PMC8748733; doi:10.1038/s41467-021-27793-5)
Supplement: Supplementary file 1 — Supporting Information [file 41467_2021_27793_MOESM1_ESM.pdf]

Supporting Information

How palladium inhibits CO poisoning during electrocatalytic formic acid oxidation and carbon dioxide reduction

Xiaoting Chen,[†] Laura P. Granda-Marulanda,[†] Ian T. McCrum,[†] and Marc T.M. Koper*[†]

[†]Leiden Institute of Chemistry, Leiden University, PO Box 9502, 2300 RA, Leiden (the Netherlands)

Experimental Details

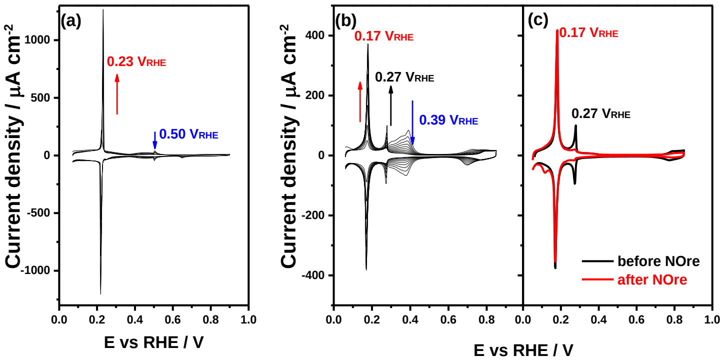

Figure S1. Cyclic voltammograms for (a) Pt(111) and (b) Pt(100) in 0.1 M H_2SO_4 + 0.1 mM $PdSO_4$, recorded in successive stages during Pd deposition experiment. Scan rate: 50 mV s^{-1}. Arrows indicate the evolution with time. (c) Stable cyclic voltammograms of Pd/Pt(100) electrode in 0.1 M H_2SO_4 before NO adsorption and reductive stripping (black line) and the same Pd/Pt(100) electrode in 0.1 M H_2SO_4 after NO adsorption and reductive stripping (red line).

Figure S1a shows the effect of the progressive accumulation of palladium on the voltammetric profile of Pt(111) electrode during the electrochemical deposition of palladium monolayer. At the shortest deposition times, the presence of palladium on the surface is reflected in the growth of a sharp adsorption state at 0.23 V_{RHE}, simultaneously with the progressive decrease of the characteristic adsorption states of Pt(111) in 0.1 M H_2SO_4. In addition, the presence of the characteristic spike of Pt(111) at 0.50 V_{RHE} strongly suggests the existence of wide Pt(111) domains. Increasing deposition cycles lead to the contributions from the Pt(111) domains around 0.50 V_{RHE} to become blocked progressively. A previous study using in situ scanning tunnelling microscopy (STM) showed an complete pseudomorphic monolayer of Pd is formed prior to bulk deposition during electrochemical deposition of Pd on Pt(111).[1] The voltammetric charge of (bi)sulfate adsorption at 0.23 V_{RHE} can be related in a quantitative way to the palladium coverage and increases to a charge value of 320 µC cm^{-2} for $Pd_{ML}Pt(111)$.[2,3]

Figure S1b shows the characteristic adsorption peak of Pt(100) electrode at 0.39 V_{RHE} progressively diminishes whereas a new sharp adsorption state appears at 0.17 V_{RHE}. The experimental result is interpreted as the progressive blocking of the Pt(100) substrate sites by a first monolayer of palladium atoms directly deposited on the Pt(100) substrate. As the deposition continues a new feature appears at 0.27 V_{RHE} while the peak at 0.39 V_{RHE} corresponding to the remaining Pt(100) unblocked sites has not been completely suppressed. The appearance of a second adsorption state at 0.27 V_{RHE} for palladium deposited on Pt(100) substrates is associated to the growth of palladium in second, third and further layers. The easiest way to obtain a Pt(100) electrode fully covered by a single palladium monolayer is to deposit palladium until all the Pt(100) sites are blocked and then remove the excess by the NO treatment described above:[4]

$$Pt(100) + Pd_{further\ layers}/Pd_{first\ layer}/Pt(100) + NO \rightarrow Pd_{first\ layer}/Pt(100)\text{-}NO + Pd_{sol}$$

in which $Pd_{first\ layer}$ means palladium adatoms in the first monolayer, $Pd_{further\ layers}$ is the second and multilayers, and Pd_{sol} represents stable palladium species in solution.

Figure S1c shows the final voltammogram in 0.1 M H_2SO_4 (red line): a characteristic peak at 0.17 V_{RHE} related to the (bi)sulfate anion adsorption on Pd monolayer is observed, the contributions assigned to the second stage of Pd deposition at 0.27 V_{RHE} and uncovered Pt(100) electrode domain at 0.39 V_{RHE} have been eliminated. The voltammogram of $Pd_{ML}Pt(100)$ is stable upon successive cycles, suggesting that the monolayer does not undergo further modification.

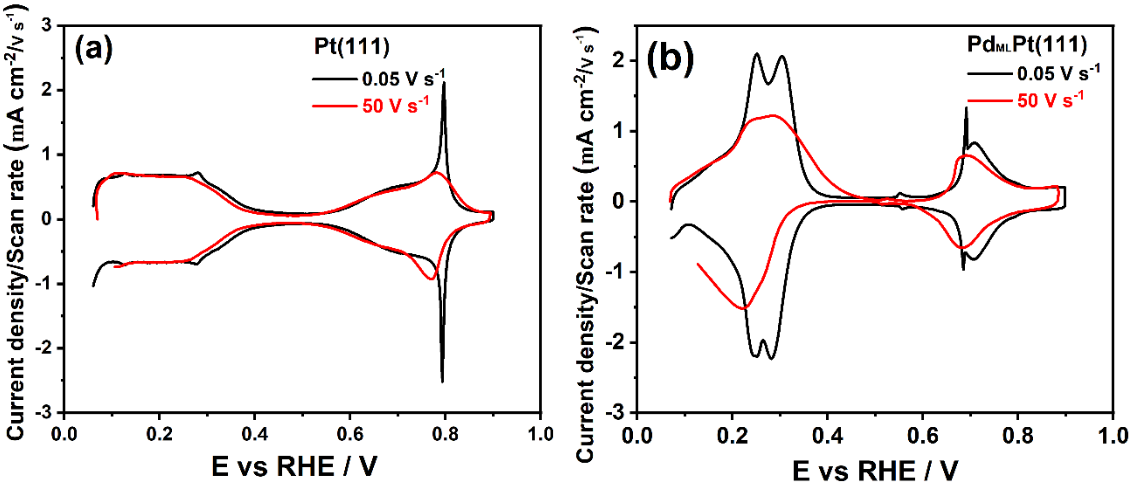

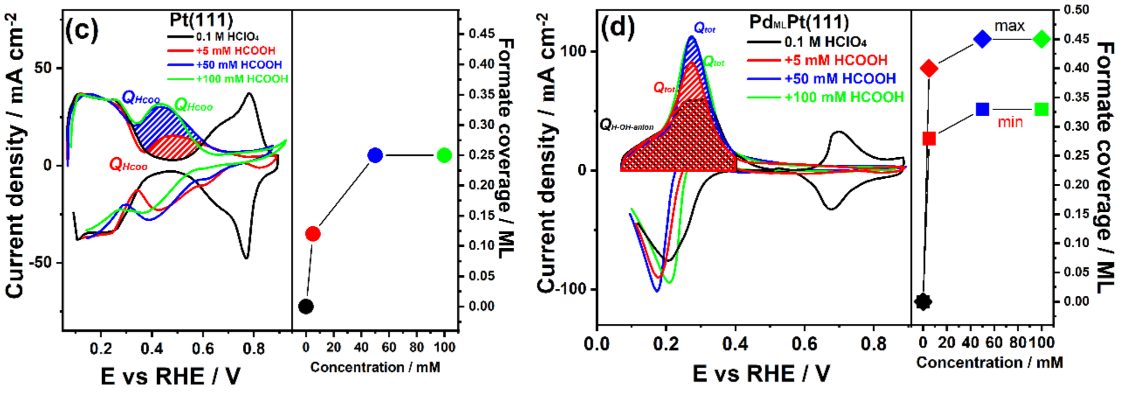

Figure S2. Voltammograms of (a) Pt(111) and (b) $Pd_{ML}Pt(111)$ electrode in 0.1 M $HClO_4$ recorded at 0.05 V s^{-1} (black line) and 50 V s^{-1} (red line), resp. Corresponding voltammograms of (c) Pt(111) and (d) $Pd_{ML}Pt(111)$ electrode in 0.1 M $HClO_4$ + *x* mM HCOOH recorded at 50 V s^{-1}, resp. The insert at right side represents the correlation between the formate coverage and formic acid concentration, resp.

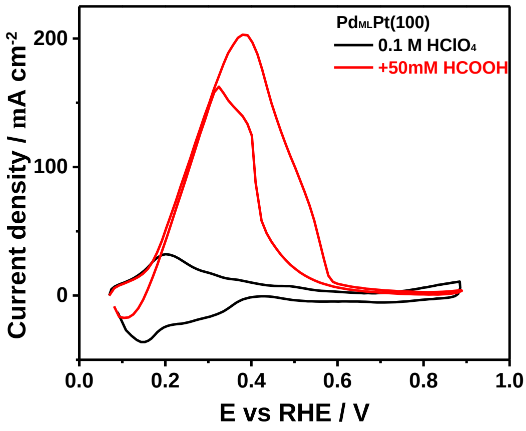

Figure S3. Voltammograms of $Pd_{ML}Pt(100)$ electrode in 0.1 M $HClO_4$ (black line) and 0.1 M $HClO_4$ + 50 mM HCOOH, recorded at 50 V s^{-1}.

Figure S4 shows the oxidation of formic acid on $Pd_{ML}Pt(100)$ electrode in 0.1 M $HClO_4$ + 50 mM HCOOH at a high scan rate of 50 V s^{-1}. The current corresponding to the oxidation of formic acid process is much larger than the current corresponding to the reversible formate adsorption/desorption so that this latter contribution cannot be separated from the voltammogram. The results suggest a much faster kinetics of formic acid oxidation reaction on $Pd_{ML}Pt(100)$ than that on $Pd_{ML}Pt(111)$ electrode.

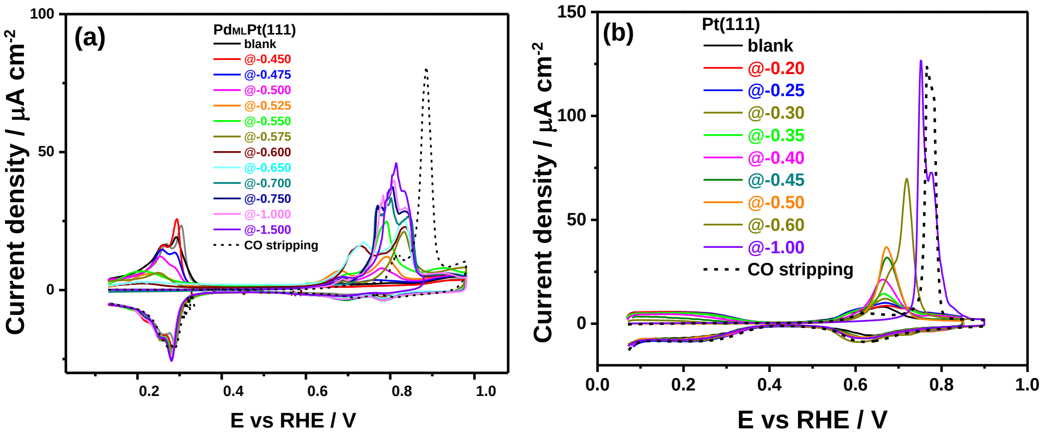

Figure S4. Voltammograms for the oxidative stripping of CO adlayer produced on (a) $Pd_{ML}Pt(111)$ and (b) Pt(111) electrode after doing CO_2 reduction at different vertex potentials in pH=3.0 (0.001M $HClO_4$/0.099M $KClO_4$) solution saturated with CO_2, recorded at 10 mV s^{-1}. A CO stripping experiment result (dashed line) of a saturated CO adlayer is performed under identical condition for comparison.

Figure S5 shows the voltammograms for the oxidative stripping of CO adlayer produced during CO_2 reduction on the $Pd_{ML}Pt(111)$ and Pt(111) electrode, resp. Figure S5a shows anodic peaks between 0.650 and 0.900 V_{RHE} are observed when doing CO_2 reduction on the $Pd_{ML}Pt(111)$ electrode by increasing the vertex potential in steps of 0.025 V from -0.475 V_{RHE}. These anodic peaks correspond to the oxidation of adsorbed CO formed during CO_2 reduction. It is reasonable to assume that there is no CO formation from CO_2 reduction on $Pd_{ML}Pt(111)$ surface at lower overpotentials than that of -0.475V_{RHE}. In Figure S4a, the production of the CO is shown to increase with increasing the overpotential for CO_2 reduction: for the overpotential of -0.475, -0.500, -0.525, -0.550, -0.575, -0.600, -0.650, -0.700 and -0.750 V_{RHE}, the coverage of CO adlayer is 0.13, 0.19, 0.22, 0.29, 0.31, 0.52, 0.58, 0.71 and 0.72 ML, resp. As can be seen from Figure S4a, the onset potential and shape of CO adlayer oxidation peak on the $Pd_{ML}Pt(111)$ electrode strongly depends on its coverage. The full CO adlayer is stripped off at 0.90 V_{RHE} (dashed line); such a high coverage is noy obtained during CO_2 reduction. The subsequent scan indicates the entire CO adlayer on $Pd_{ML}Pt(111)$ electrode was stripped in a single positive-going sweep and retains the well-defined hydrogen adsorption and anion desorption features in the low-potential region.

In the case of the Pt(111) electrode, reducing CO_2 to adsorbed CO starts at -0.25 V_{RHE} and the surface is poisoned when the negative vertex potential reaches -0.60 V_{RHE}. Figure S4b shows that with increasing overpotential for CO_2 reduction on Pt(111), the formation of the CO increases: for the overpotential of -0.25, -0.30, -0.35, -0.40, -0.50, -0.60 and -1.0 V_{RHE}, the coverage of CO adlayer is 0.23, 0.37, 0.37, 0.47, 0.59, 0.63 and 0.66 ML, resp. The subsequent scan shows the recovery of the Pt(111) surface after the CO adlayer oxidation.

Computational Details

Free energies calculations

The adsorption free energies of *H, *CO, *OCHO and *COOH were calculated from formic acid in solution, (HCOOH (aq)) for the formic acid oxidation reaction, and from carbon dioxide in gas-phase, (CO_2 (g)) for the CO_2 reduction reaction. Below we show how the free energies are calculated in both cases.

Formic Acid oxidation

The following chemical equations show how the adsorption free energies of the different adsorbates are calculated for formic acid oxidation.

$$HCOOH(aq) + * \rightarrow *H + CO_2(g) + H^+ + e^- \quad (1)$$

$$HCOOH(aq) + * \rightarrow *OCHO + H^+ + e^- \quad (2)$$

$$HCOOH(aq) + * \rightarrow *COOH + H^+ + e^- \quad (3)$$

$$HCOOH(aq) + * \rightarrow *CO + H_2O(l) \quad (4)$$

where, * represents the adsorption site. The free energies of adsorption are then calculated as shown below

$$G_{*H}^{ads} = G_{*H} + G_{CO_2(g)} + \frac{1}{2}G_{H_2(g)} - G_{HCOOH(aq)} - G_* \quad (5)$$

$$G_{*OCHO}^{ads} = G_{*OCHO} + \frac{1}{2}G_{H_2(g)} - G_{HCOOH(aq)} - G_* \quad (6)$$

$$G_{*COOH}^{ads} = G_{*COOH} + \frac{1}{2}G_{H_2(g)} - G_{HCOOH(aq)} - G_* \quad (7)$$

$$G_{*CO}^{ads} = G_{*CO} + G_{H_2O(l)} - G_{HCOOH(aq)} - G_* \quad (8)$$

Each free energy is calculated as G = EDFT + ZPE + TS, where EDFT is the energy obtained from the DFT calculation at 0K, ZPE is the zero-point energy determined from the vibrational frequencies obtained using the harmonic-oscillator approximation. The TS is the temperature at T= 298.15 K times the entropy (S) term containing i) all the contributions (translational, rotational and vibrational) for the free energies of species in the

gas phase and is taken from standard thermodynamic tables[5] and ii) for the free energies of adsorbed species containing vibrational contributions. The free energy of the surface, G_*, is the energy from DFT at 0 K. The free energies of CO_2(g) and CO(g) are corrected by -0.19 and 0.24 eV, these corrections come from the difference between the experimental standard free energy of formation ΔG°_{Exp} and the DFT formation energyΔG°_{DFT}, as PBE does not describe well their formation energies.[6–8]

Free energy of solution phase HCOOH (aq)

The solution phase free energy of HCOOH (aq) was calculated using the SHE equilibrium redox potential of CO_2 (g) + $2H^+$ + $2e^-$ → HCOOH (aq), E° = -0.11 V vs SHE,[9] where the free energy of CO_2 (g) is calculated within DFT. This was used to solve for the standard free energy of formic acid in solution phase, $\Delta G^{\circ}_{HCOOH}(aq)$ and ultimately to solve for the aqueous free energy at the experimental conditions, see below.

$$\Delta G^{\circ}_{HCOOH}(aq) = G^{\circ}_{CO_2(g)} + 2G^{\circ}_{H^+} - 2|e|U \quad (9)$$

where U = E° = -0.11 V,[9] and $G^{\circ}_{H^+} = \frac{1}{2} G_{H_2(g)}$ at 0 V in the SHE scale.

The standard solution phase free energy, $\Delta G^{\circ}_{HCOOH}(aq)$, was then used to obtained the free energy of formic acid, ΔG_{HCOOH}, at the experimental conditions of 0.1M $HClO_4$ and 50 mM HCOOH, following the Nernst equation.

$$\Delta G_{HCOOH} = \Delta G^{\circ}_{HCOOH}(aq) + k_b T ln(Cf) \quad (10)$$

Cf is the actual concentration of HCOOH and is obtained using the pKa of formic acid, 3.94 and the starting concentrations of $HClO_4$ and HCOOH.

The free energies of the protons and electrons as expressed in the adsorption reactions, equations 1 to 4, were calculated using the computational hydrogen electrode (CHE) model.[10]

Formate coadsorption with *H, *CO and *COOH

To investigate the effect of formate coverage on the free energy of adsorption of *H, *CO and *COOH we calculated the energies from HCOOH (aq) and n molecules of formate *OCHO adsorbed on the surface. For example, the adsorption of *COOH at different formate coverages is calculated using the following chemical reaction

$$HCOOH(aq) + *nOCHO \rightarrow *[COOH - nOCHO] + H^+ + e^- \quad (11)$$

and its free energy of adsorption

$$G^{ads}_{*[COOH-nOCHO]} = G_{*[COOH-nOCHO]} + \frac{1}{2}G_{H_2(g)} - G_{HCOOH(aq)} - G_{*nOCHO} \tag{12}$$

with n = 1-3 using the 3x3 unit cell representing coverages of 0.11ML to 0.33 ML and with n = 1 using the 2x2 unit cell to represent coverages of 0.25 ML. *[COOH-nOCHO] means both COOH and n OCHO are adsorbed in the same unit cell.

CO_2 Reduction

Similarly, the adsorption energetics were calculated for the reduction reaction, i.e. the production of formic acid from CO_2. This time the reference state is based on CO_2 (g), protons, and electrons. All the gas-phase corrections for CO_2(g) and CO (g) are included as discussed above, and the CHE model is used for the coupled proton and electron transfer.

$$H^+ + e^- + * \rightarrow *H \tag{13}$$

$$CO_2(g) + H^+ + e^- + * \rightarrow *OCHO \tag{14}$$

$$CO_2(g) + H^+ + e^- + * \rightarrow *COOH \tag{15}$$

$$CO_2(g) + 2H^+ + 2e^- + * \rightarrow *CO + H_2O(l) \tag{16}$$

The free energies of adsorption are then calculated as shown below.

$$G^{ads}_{*H} = G_{*H} - \frac{1}{2}G_{H_2\,(g)} - G_* \tag{17}$$

$$G^{ads}_{*OCHO} = G_{*OCHO} - G_{CO_2(g)} - \frac{1}{2}G_{H_2(g)} - G_* \tag{18}$$

$$G^{ads}_{*COOH} = G_{*COOH} - G_{CO_2(g)} - \frac{1}{2}G_{H_2(g)} - G_* \tag{19}$$

$$G^{ads}_{*CO} = G_{*CO} + G_{H_2O(l)} - G_{CO_2(g)} - G_{H_2(g)} - G_* \tag{20}$$

The limiting potentials for the first protonation step during CO_2 reduction reaction to *COOH are calculated at the potential when the G^{ads}_{*COOH}= 0 as show in in Eq.27.

$$CO_2 + (H^+ + e^-) + * \rightarrow * COOH \quad (21)$$

$$G_{*COOH}^{ads} = G_{*COOH} - G_{CO_2(g)} - \frac{1}{2} G_{H_2(g)} - G_* + |e|U \quad (22)$$

$$U = \frac{G(CO_2\ g) + \frac{1}{2} G\left(H_2(g)\right) + G * COOH + G(*)}{1\ |e|} \quad (23)$$

Table S1 shows the free energies adsorption for *COOH , the precursor of *CO, with H-up and H-down (see Figure S6), calculated from HCOOH (aq), Eq. (7, and from CO_2 (g), Eq. (19, at 1/9 ML coverage on $Pd_{ML}Pt(111)$, Pt(111) and Pd(111).

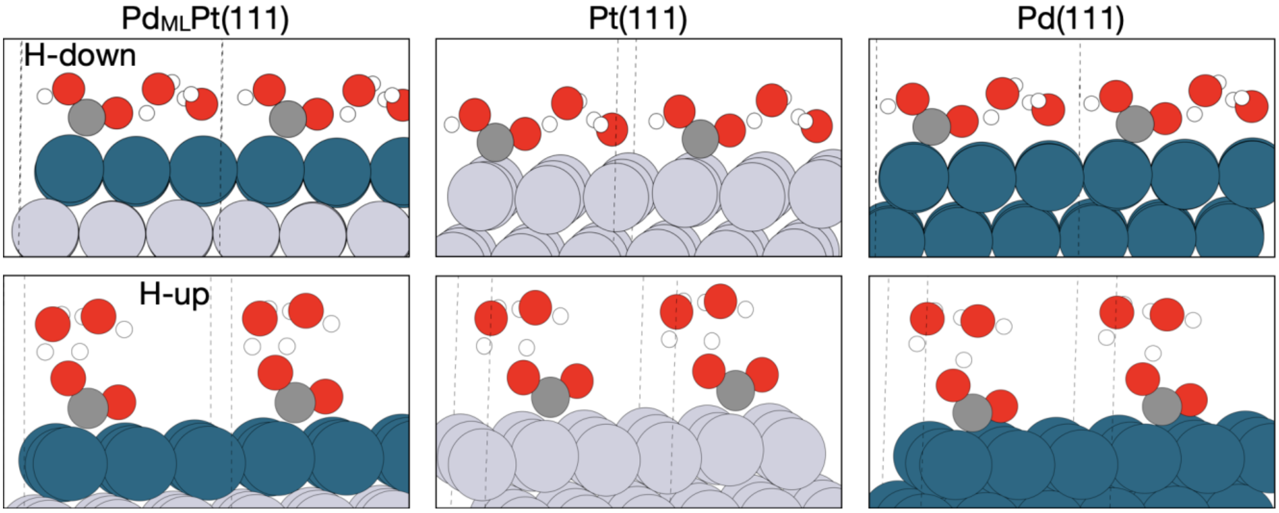

Figure S5: Illustration of *COOH solvated with two explicit water molecules with hydrogen down (H-down) and hydrogen up (H-up) configuration on the $Pd_{ML}Pt(111)$, Pt(111), and Pd(111) surfaces. The boundary of the unit cell is delineated by the vertical dashed line, and each figure shows two-unit cells of 3x3.

Table S1: Free energies of adsorption for solvated *COOH with H-up and H-down configuration at 1/9 ML coverage calculated for formic acid oxidation reaction, and for CO_2 reduction reaction, where HCOOH (aq) or CO_2 (g) are the reference state respectively. Energies are in eV.

	$Pd_{ML}Pt(111)$	Pt(111)	Pd(111)
COOHup-sol from HCOOH (aq)	-0.15	-0.48	-0.10
COOHdown-sol from HCOOH (aq)	-0.37	-0.63	-0.29
COOHup-sol from CO_2 (g)	-0.003	-0.34	0.04
COOHdown-sol from CO_2(g)	-0.23	-0.49	-0.15

Effect of hydrogen coverage on the free energy of *COOH

We investigated the influence of the hydrogen coverage on the adsorption energy of *COOH-sol, the precursor of *CO formation, on the three different surfaces. The energy of *COOH-sol is described further below in the solvation effects section. Basically, the solvation energy is added to the free energies of the systems in vacuum. The free energies were calculated following the equations below, *nH represents the surface with n number of adsorbed hydrogens per unit cell. The hydrogen coverages investigated were 1/3 ML and 1ML.

From Figure S6 we can see that the effect of hydrogen coverage on the adsorption energy of *COOH-sol is a decrease in its binding energy, as expected due to repulsion interactions, but comparing between the three surfaces *COOH-sol adsorbs on Pt(111) stronger.

$$CO_2 + *nH + H^+ + e^- \rightarrow *[COOH - nH] \quad (24)$$

$$G^{ads}_{*[COOH-nH]} = G_{*[COOH-nH]} - G_{CO_2} - G_{*nH} - \frac{1}{2}G_{H_2(g)} \quad (25)$$

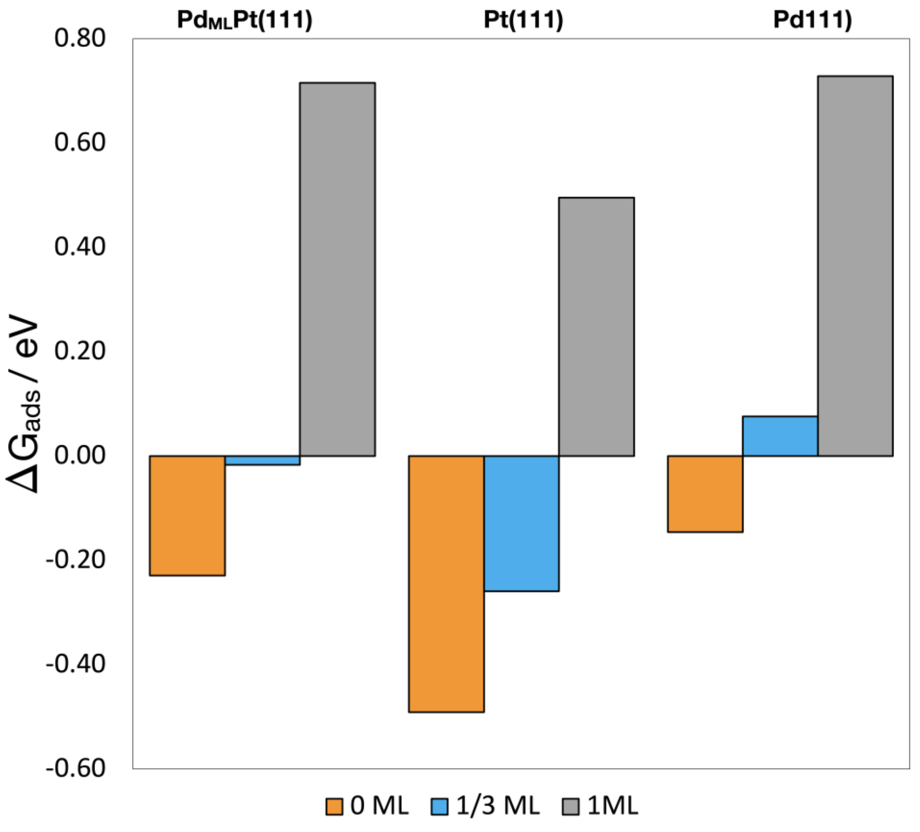

Figure S6: Free energy of adsorption of *COOH in the absence (orange) and presence of different coverages of co-adsorbed hydrogen 1/3 ML (blue) and 1 ML (grey) on $Pd_{ML}Pt(111)$, Pt(111) and Pd(111) at 0 V vs RHE , the solvation energy is added to *COOH in all cases as explained in the solvation effects section.

Solvation effects

Solvation effect on *COOH

We consider the effect of solvation on the adsorption of *COOH by solvating *COOH with 2 explicit water molecules, named as *COOH-sol. Adsorbed water bilayer and solution-phase formic acid were used as the reference states, see Eq. (**26** for formic acid oxidation, while for the reduction reaction we use adsorbed water, CO_2 (g), and protons and electrons, Eq. (**27**. The adsorbed water reference state is the adsorbed water in the ice-like structure in a 3x3 unit cell, while in 2x2 unit cell we used a reference state of 4 adsorbed hydrogen bonded water molecules. The following equations correspond to n=6 in the 3x3 unit cell and n=4 in the 2x2 unit cell.

$$HCOOH(aq) + \ n * H_2O \rightarrow * [COOH - 2H_2O] + (n-2)H_2O(l) \ + \ H^+ + \ e^- \qquad (26)$$

$$CO_2(g) + \ n * H_2O + \ H^+ + \ e^- \rightarrow \ * [COOH - 2H_2O] + (n-2)H_2O(l) \qquad (27)$$

The solvation energy of adsorbed *COOH, Ω_{*COOH}, is the difference between the non-solvated and solvated free energies of *COOH. That difference gives Eq. (28 and the solvation energy is Eq. (29

$$* COOH \ + \ n * H_2O \rightarrow * [COOH - 2H_2O] + (n-2)H_2O(l) \ + \ * \qquad (28)$$

$$\Omega_{*\mathrm{COOH}} = \ G_{*[COOH-2H_2O]} + \ (n-2)G_{H_2O(l)} + G_* - G_{*COOH} - n\,G_{*H_2O} \qquad (29)$$

The solvation energy, $\Omega_{*\mathrm{COOH}}$, calculated here is an estimate to capture the effect of solvation of coabsorbed *COOH with *OCHO, that is *[COOH-nOCHO], and to capture the effect of coadsorbed *COOH with hydrogen *[COOH-nH]. To account for such solvation effects, the solvation energy is added to the final free energy of adsorption as, $G^{ads}_{*[COOH-nOCHO]} + \ \Omega_{*COOH}$, and $G^{ads}_{*[COOH-nH]} + \ \Omega_{*COOH}$ and represents an upper bound estimate of the solvation effect.

Table S2 shows the solvation energies determined for *COOH with H-up and H-down configuration at both 1/9 ML and 1/4 ML coverages on $Pd_{ML}Pt(111)$, Pt(111) and Pd(111) surfaces. We determine 3 different solvation energies following Eq. (29. For example, H-down vac to Hup-sol, means that the reference state (*COOH) is adsorbed *COOH with hydrogen down configuration in vacuum (H-down vac), and the solvated *COOH is with hydrogen in the up configuration (Hup-sol). At high coverages 1/4 ML *COOH, higher stabilization (more negative solvation energies) is achieved for the *COOH with H up configuration, while at low coverages higher stabilization is achieved for H down-vac to H down-sol. The reason for this might be more predominant repulsive interactions than solvation stabilization at higher coverages than at low coverages.

Table S2: Calculated solvation energies for *COOH adsorbate on $Pd_{ML}Pt(111)$, Pt(111), Pd(111), at 1/9 ML and 1/4 ML coverages following Eq. (29). The solvation energies where calculated following different positions of the hydrogen on *COOH, where the H of the OH group could be on the H-down position or up.

3x3-111 (1/9 ML)	$Pd_{ML}Pt(111)$	Pt(111)	Pd(111)
H down vac to H up-sol	-0.30	-0.47	-0.35
H up vac to H up-sol	-0.38	-0.59	-0.46
H down vac to H down-sol	-0.53	-0.62	-0.54
2x2-111 (1/4 ML)	$Pd_{ML}Pt(111))$	Pt(111)	Pd(111)
H down vac to H up-sol	-0.17	-0.20	-0.28
H up vac to H up-sol	-0.33	-0.44	-0.42
H down vac to H down-sol	-0.13	-0.12	-0.29

In summary, *COOH is preferably adsorbed with a H-down type configuration at least up to 0.25 ML coverage of formate. Once the coverage of formate is increased to 0.33ML, *COOH with H-up configuration is more favorable. Therefore, in the coadsorbed system, where *COOH is co-adsorbed with 0.33 ML of *OCHO, we corrected for solvation with the solvation energy determined for the *COOH with H-up configuration.

*COOH coadsorbed with 1ML of hydrogen prefers to adsorb with H down configuration. For Pd and $Pd_{ML}Pt(111)$ it is ~0.2 eV more favoarble than H-down, while for Pt (111) is more favorable by ~ 0.04 eV. Therefore, we corrected the energy of the coadsorbed system with the solvation energy determined for *COOH with H-down configuration.

Again, these solvation corrections are an upper bound estimation of the solvation effect on *COOH.

Solvation effects on *OCHO

Formate retains partial negative charge upon adsorption; to assess whether or not adsorbed formate might benefit from solvation, we determine its solvation energy with 1, 2, and 3 explicit water molecules following the assessment described in ref [11]. Briefly, the adsorbate in question can benefit from solvation via hydrogen bonding if the difference between solvation energy with (n) number of water molecules and (n-1) is more negative than the water-self solvation energy on that surface, $\Omega^{n}–\Omega^{n-1} \leq \Omega\ H_2O$, (where, Ω, refers to solvation energy). The water-self solvation energy was calculated as the difference in free energy between one water molecule at 1/9 ML and a water molecule within the water bilayer at 2/3 ML coverage. For Pt(111) that difference is -0.29 eV, for $Pd_{ML}Pt(111)$ it is -0.18 eV and for Pd it is -0.14 eV. From column Δ 2 w-1w in Table S3, the difference in solvation energies of formate with two and one water molecules is higher than the water self-solvation, on all surfaces respectively, suggesting that solvation with one water molecule is enough. This solvation energy is an upper bound estimation of the solvation effect on formate.

The reference state to calculate the solvation energy is the adsorbed water bilayer in the 3x3 (111) unit cell, at 2/3 ML coverage, and the solvation energy is the difference between the free energy of the solvated formate and the non-solvated formate, Ω_{*OCHO}.

$$* \text{OCHO} + 6 \ * H_2\text{O} \rightarrow * [\text{OCHO} - 1H_2O] + 5 \ H_2\text{O} \ (l) \ +* \quad (30)$$

$$\Omega_{*\text{OCHO}} = G_{*[OCHO-1H_2O]} + 5G_{H_2O(l)} + G_* - G_{*OCHO} - 6\,G_{*H_2O} \quad (31)$$

Table S3: Calculated solvation energies for adsorbed formate at 1/9 ML with 1, 2, and 3 water molecules. Last two columns show the difference between, 2 H_2O molecules (2w) and 1 H_2O molecules, and between 3 H_2O (3w) and 2 H_2O molecules.

Surface	1 H_2O	2 H_2O	3 H_2O	Δ 2 w-1w	Δ 3 w-2w
$Pd_{ML}Pt$ (111)	-0.50	-0.27	-0.24	0.23	0.03
Pt (111)	-0.39	-0.23	--	0.16	--
Pd (111)	-0.51	-0.25	-0.24	0.26	0.01

Dipole moments and Bader partial charges

Bader partial charge analysis was performed with the Atoms in Molecules, AIM, Bader analysis,[12,13] using the Bader program from Henkelman's group.[14]

Table S4: Dipole moments and Bader partial charges for various adsorbates on $Pd_{ML}Pt(111)$, Pt(111) and Pd(111) at 1/9 ML coverage unless specified otherwise. [a] Total partial Bader charge of hydrogens adsorbed on the surface on the fcc sites, and [b] partial Bader charge of only *COOH.

	Δμ / e*Å			|q|/ e^-		
adsorbate	$Pd_{ML}Pt(111)$	Pt(111)	Pd(111)	$Pd_{ML}Pt(111)$	Pt(111)	Pd(111)
H-fcc	0.02	-0.01	0.01	-0.09	-0.03	-0.08
H-top	0.03	-0.03	0.02	0.00	0.04	0.00
CO	0.18	-0.03	0.17	-0.23	-0.01	-0.22
OCHO	-0.06	-0.23	-0.07	-0.50	-0.40	-0.48
COOH- H up	-0.24	-0.16	-0.25	-0.22	-0.16	-0.19
COOH- H down	0.06	0.21	0.06	-0.14	0.01	-0.10
COOH-Hup sol	-0.23	-0.18	-0.29	-0.20	-0.12	-0.17
COOH-Hdown sol	0.06	-0.35	-0.17	-0.15	-0.08	-0.11

OCHO-sol	-0.25	-0.41	-0.24	-0.58	-0.39	-0.39
1/3 ML Hads-fcc	0.06	-0.03	0.04	-0.25	-0.06	-0.21
1 ML Hads-fcc	0.09	-0.17	0.06	-0.56	-0.07	-0.51
[a]1/3 ML *H in *COOH	0.12	0.18	0.15	-0.19	-0.05	-0.19
[a] 1 ML *H in *COOH	0.24	0.05	0.20	-0.61	-0.05	-0.48
[b]*COOH in 1/3 ML *H	--	--	--	-0.08	-0.01	-0.04
[b] *COOH in 1 ML *H	--	--	--	0.03	0.02	0.07

Work functions

Work functions, Φ , were determined as, $\Phi = V - E_{Fermi}$, the difference between the Fermi energy and the one electron potential in vacuum.

Table S5: Calculated work function for the bare surfaces, $Pd_{ML}Pt(111)$, Pt(111) and Pd(111), and at different hydrogen coverages.

	Φ/ eV		
	$Pd_{ML}Pt(111)$	Pt (111)	Pd (111)
Bare	5.14	5.74	5.29
1/9 ML	5.20	5.71	5.32
1/3 ML	5.33	5.64	5.37
1ML	5.45	5.20	5.42

References

1. Wakisaka, M., Asizawa, S., Uchida, H. & Watanabe, M. In situ STM observation of morphological changes of the Pt(111) electrode surface during potential cycling in 10 mM HF solution. *Phys. Chem. Chem. Phys.* **12**, 4184–4190 (2010).
2. Álvarez, B., Climent, V., Rodes, A. & Feliu, J. M. Anion adsorption on Pd–Pt(111) electrodes in sulphuric acid solution. *J. Electroanal. Chem.* **497**, 125–138 (2001).

3. Soriaga, M., Stickney, J., Bottomley, L. & Kim, Y. *Thin Films: Preparation, Characterization, Applications*. (Springer US, 2002).

4. Álvarez, B., Berná, A., Rodes, A. & Feliu, J. M. Electrochemical properties of palladium adlayers on Pt(100) substrates. *Surf. Sci.* **573**, 32–46 (2004).

5. Chase, Jr., M. W. *et al.* NIST-JANAF Thermochemical Tables. *J. Phys. Chem. Ref. Data* **Suppl. 1 to Vol. 14**, 1856 (1985).

6. Peterson, A. A., Abild-Pedersen, F., Studt, F., Rossmeisl, J. & Nørskov, J. K. How copper catalyzes the electroreduction of carbon dioxide into hydrocarbon fuels. *Energy Environ. Sci.* **3**, 1311–1315 (2010).

7. Christensen, R., Hansen, H. A. & Vegge, T. Identifying systematic DFT errors in catalytic reactions. *Catal. Sci. Technol.* **5**, 4946–4949 (2015).

8. Granda-Marulanda, L. P. *et al.* A Semiempirical Method to Detect and Correct DFT-Based Gas-Phase Errors and Its Application in Electrocatalysis. *ACS Catal.* **10**, 6900–6907 (2020).

9. Bratsch, S. G. Standard Electrode Potentials and Temperature Coefficients in Water at 298.15 K. *J. Phys. Chem. Ref. Data* **18**, 1–21 (1989).

10. Nørskov, J. K. *et al.* Origin of the Overpotential for Oxygen Reduction at a Fuel-Cell Cathode. *J. Phys. Chem. B* **108**, 17886–17892 (2004).

11. Rendón-Calle, A., Builes, S. & Calle-Vallejo, F. Substantial improvement of electrocatalytic predictions by systematic assessment of solvent effects on adsorption energies. *Appl. Catal. B Environ.* **276**, 119147 (2020).

12. Bader, R. F. W. *Atoms in molecules: a quantum theory*. (Clarendon Press, 1994).

13. Bader, R. F. W. Atoms in molecules. *Acc. Chem. Res.* **18**, 9–15 (1985).

14. Henkelman, G., Arnaldsson, A. & Jónsson, H. A fast and robust algorithm for Bader decomposition of charge density. *Comput. Mater. Sci.* **36**, 354–360 (2006).
